# Clinical and functional characterization of a novel *TNFRSF9* variant causing immune dysregulation with predisposition to EBV-driven lymphomagenesis

**DOI:** 10.3389/fimmu.2025.1605221

**Published:** 2025-08-06

**Authors:** Peiwei Zhao, Kailan Chen, Li Yang, Chunhui Wan, Lei Zhang, Sukun Luo, Xuelian He

**Affiliations:** ^1^ Precision Medical Center, Wuhan Children’s Hospital (Wuhan Maternal and Child Healthcare Hospital), Tongji Medical College, Huazhong University of Science & Technology, Wuhan, China; ^2^ WuhanChildren’s Hospital (Wuhan Maternal and Child Healthcare Hospital), Tongji Medical College, Huazhong University of Science & Technology, Clinical Medical Research Center for Birth Defect Prevention and Treatment in Wuhan, Wuhan, China; ^3^ Department of Pediatric Oncology, Wuhan Children’s Hospital (Wuhan Maternal and Child Healthcare Hospital), Tongji Medical College, Huazhong University of Science & Technology, Wuhan, China; ^4^ Laboratory of Hematology, Wuhan Children’s Hospital (Wuhan Maternal and Child Healthcare Hospital), Tongji Medical College, Huazhong University of Science & Technology, Wuhan, China

**Keywords:** *TNFRSF9*, immunodeficiency, lymphoproliferation, EBV viremia, NF-κB, AKT

## Abstract

**Introduction:**

The *TNFRSF9* gene encodes the costimulatory receptor CD137, also known as 4-1BB, which plays a critical role in sustaining effective cytotoxic T-cell responses. Variants in the *TNFRSF9* gene are associated with an extremely rare autosomal recessive primary immunodeficiency disorder characterized by recurrent sinopulmonary infections and EBV-induced lymphoproliferation.

**Methods:**

We report a case siblings exhibiting EBV viremia, recurrent respiratory infections, and Burkitt lymphoma. Whole-exome sequencing (WES) was performed. Sanger sequencing was used to validate the variants. In vitro functional study was performed by western blot, flow cytometry assays and luciferase assays.

**Results:**

Genetic analysis identified a novel missense variant in the *TNFRSF9* gene (NM_001561.5: c.359G>C, p.C120S). Functional analysis in vitro demonstrated that this variant decreased the expression of TNFRSF9 both mRNA and protein levels. Western blot analysis revealed a significant decrease in phosphorylated-AKT. Luciferase assays showed that the p.C120S variant diminished the activity of the NF-κB pathway. Immunophenotyping of the patient’s peripheral blood revealed a significant reduction in CD27+ memory B cells, which are critical for long-term humoral immunity. Additionally, there was a notable decrease in IFN-γ secretion in CD8+ T cells, suggesting impaired cytotoxic T-cell function. These findings align with the clinical presentation of immunodeficiency and lymphoproliferation observed in the patients. We also reviewed 9 previously reported patients with homozygous or compound heterozygous *TNFRSF9* variants. The clinical manifestations among these patients were highly heterogeneous, ranging from asymptomatic to malignancies.

**Discussion:**

In summary, we identified a novel *TNFRSF9* variant associated with immunodeficiency and lymphoproliferation, supported by functional evidence demonstrating its impact on gene expression, AKT and NF-κB signaling pathways, and immune cell function. Our findings expand the mutation spectrum of the *TNFRSF9* gene and provide new insights into the molecular mechanisms underlying this rare immunodeficiency disorder.

## Introduction

Epstein-Barr Virus (EBV), a ubiquitous gamma herpesvirus, infects over 90% of the global population and establishes life-long latency, primarily in B cells, though it can also infect epithelial, T, and NK cells (1). In immunocompetent individuals, primary EBV infection is often asymptomatic or manifests as self-limiting infectious mononucleosis (IM), with EBV-induced B-cell proliferation effectively controlled by T and NK cells. However, in immunodeficient patients, impaired immune surveillance leads to uncontrolled EBV-driven lymphoproliferation, resulting in severe complications such as hemophagocytic lymphohistiocytosis (HLH), persistent viremia, and malignancies of B-, T-, or NK-cell origin (2, 3). Given the critical role of T and NK cells in controlling EBV infection and related diseases, molecules that enhance their function are of particular interest.

*TNFRSF9* (CD137/4-1BB), a member of the tumor necrosis factor receptor (TNFR) superfamily, encodes a critical costimulatory molecule that promotes CD8+ T-cell proliferation, survival, and cytolytic activity (4). The human 4-1BB protein, a 255-amino acid type I transmembrane protein, consists of an N-terminal signal peptide, an extracellular domain with four cysteine-rich domains (CRDs), a transmembrane region, and a cytoplasmic signaling domain containing a TRAF-binding motif (5). The CRDs, characterized by intra-domain disulfide bonds, form elongated structures that facilitate ligand binding and receptor aggregation, with CRD2 and CRD3 forming the ligand-binding domain. *TNFRSF9* is primarily expressed on activated T cells and its ligand, 4-1BBL (TNFSF9), a type II transmembrane protein of the TNF superfamily, is predominantly expressed on antigen-presenting cells (APCs) such as B cells, macrophages, and dendritic cells. Ligand binding induces receptor clustering and recruitment of TRAF adaptor proteins (TRAF1 and TRAF2), activating NF-κB, PI3K/AKT, and MAPK signaling pathways. Activation of *TNFRSF9* enhances the production of IFN-γ and perforin, key mediators of CD8+ T cells cytolytic function (6, 7). The essential role of *TNFRSF9* in CD8+ T-cell immunity is further underscored by studies in *TNFRSF9* deficient mice, which exhibited impaired IFN-γ production and reduced cytolytic CD8+ T-cell effector function, highlighting its importance in immune responses (8).

Variants in *TNFRSF9* cause immunodeficiency 109 with lymphoproliferation (IMD109, OMIM #620282), a rare autosomal recessive disorder characterized by immunodeficiency, recurrent infection, hypogammaglobulinemia, EBV viremia, and EBV-induced lymphoproliferative disorders or lymphoma (9). First described by Alosaimi, et al. in 2019, IMD109 is extremely rare, with only 9 reported patients to date (4, 9–11). Here, we present a case from a Chinese family featuring Burkitt lymphoma, recurrent respiratory infections, conjunctivitis, and EBV viremia, harboring a homozygous *TNFRSF9* variant (c.359G>C, p.C120S). *In vitro* studies demonstrated that this variant impairs NF-κB and AKT signaling pathways, providing mechanistic insights into its pathogenic role. Our findings expand the genetic and clinical spectrum of *TNFRSF9*-related disease and include a comprehensive review of the clinical phenotypes and genotypes of all reported patients with *TNFRSF9* variants.

## Materials and methods

### Subjects

A patient diagnosed with lymphoma 4 years ago was enrolled in this study due to recurrent respiratory infections and conjunctivitis persisting for over one year. Following informed consent from the parents of the patient, peripheral blood samples were collected from the patient and his parents for biochemical and genetic analyses. Genomic DNA and RNA were extracted from whole blood using the QIAamp Blood DNA mini kit (Qiagen) and Trizol reagent (Invitrogen) respectively, following standard protocols and manufacturer instructions. The patient had a 16-year-old sibling who succumbed to nasopharyngeal carcinoma (NPC). Since the age of 7, the patient exhibited recurrent infections and EBV viremia. This study was approved by the Ethics Committee of Wuhan Children’s Hospital, Tongji Medical College, and Huazhong University of Science & Technology (Approval No. 2020R006-E5).

### Genetic analysis

Trio whole exome sequencing (WES) and subsequent data analysis were conducted by a third-party medical testing laboratory (Chigene (Beijing) Translational Medical Research Center, China), as previously described. The identified candidate genetic variant was verified using Sanger sequencing with custom-designed primers: *TNFRSF9*-F (GTCCCTGTCCTCCAAATAGTTTC) and *TNFRSF9*-R (GAACAGTTTGTCCAGGGTCGAC) using an ABI 3730XL DNA sequencer. Conservative amino acid analysis was conducted by MEGA software. PyMOL software was employed for 3D structural visualization.

### *TNFRSF9* plasmid construction and cell transfection

The *TNFRSF9* target sequence was amplified from PBMCs with specific primers. The wild-type *TNFRSF9* (WT-*TNFRSF9*) plasmid was constructed by inserting the target fragment into the pcDNA3.1 (+) vector using XhoI and EcoRI restrictions sites. Mutated plasmids (C120S-*TNFRSF9* and G109S-*TNFRSF9*, the latter serving as a positive control) were generated via the site-directed mutagenesis using overlap PCR. All constructs were verified by Sanger sequencing. We also constructed fusion proteins of GFP-TNFRSF9 (WT, G109S and C120S) using EGFP-C1 plasmid. 293T cells cultured in DMEM supplemented with 10% fetal bovine serum at 37°C under 5% CO_2_, were transfected with 1 μg of plasmids using Lipfectamine 3000 (Invitrogen) according to the manufacturer’s protocols. Fluorescence intensity was analyzed by flow cytometry to evaluate cell transfection efficiency.

### Quantitative real-time PCR

Total RNA was isolated from 293T cells transfected with wild-type, mutant *TNFRSF9*, or control plasmids. cDNA was synthesized using reverse transcriptase (TAKARA, Dalian) and random hexamer primers (Invitrogen). Quantitative real-time PCR was performed using the SYBR Green PCR kit (TAKARA, Dalian), with *GAPDH* as the internal control.

### Western blotting

Western blotting was performed as previously described. Membranes were incubated with primary antibodies against Flag (Proteintech, 66008-4-Ig), AKT (Cell signaling technology, 4685), Phosphorylated AKT (pS473, Cell signaling technology, 4060), and GAPDH (Proteintech, 60004-1-Ig), followed by incubation with secondary antibodies. Protein bands were visualized using an ECL peroxidase substrate. These experiments were repeated three times and the protein expression level was quantified by gray value using Image J software.

### Luciferase reporter assays

293T cells were seeded in 24-well plates (2 × 10^5^ cells/well) and co-transfected with 300 ng of pcDNA3.1+, *TNFRSF9*-expression vector (WT, C120S and G109S) as needed, 150 ng of pNFκB-luc (Bayotime, Shanghai, China), and 100 ng of pRL-TK control plasmid (Promega, Madison, WI). After 36h, cells were lysed, and luciferase activity was measured using the Dual-Luciferase Reporter Assay System (Promega, Madison, WI). Experiments were performed in triplicate.

### Flow cytometry assays

Lithium-heparin anticoagulated blood samples from the patient and his mother were analyzed for T cell function. Blood samples (100μL) were mixed with 400μL of 1640 medium and stimulated with 1μL stimulator containing PMA, Ionomycin, and brefeldin A (BD Biosciences, #550583) for 4 hours at 37°C under 5% CO2. Cells were stained with CD3-FITC (BD Biosciences, clone SK7), CD19-PE (BD Biosciences, clone 4G7), CD27-PE-CY7 (Biolegend, clone M-T271), and CD8-APC-CY7 (BD Biosciences, SK1) for 15 minutes, followed by fixation with 100μL solution A (BD IntraSureTM Kit, #641776) for 15 minutes. After that, the precipitated cells were centrifuged by hemolysis and stained with IFN-γ-APC (BD Biosciences, clone 25723.11) in 50ul solution B (BD IntraSureTM Kit, #641776) for 15 minutes. Data were acquired using FACSCanto™ II (BD Bioscience) and analyzed by FlowJo software.

### Statistics methods

Statistical analyses were performed using Graphpad Prism 5. Experiments, including luciferase activity, WB experiments was repeated three times, and data are reported as the mean ± SD. A Student’s t-test was used to compare two groups, and a one-way analysis of variance (ANOVA) was used when comparing three or more groups and statistical significance is indicated by *P<0.05; **P<0.01, ***P<0.001.

## Results

### Case presentation

**Figure 1:**
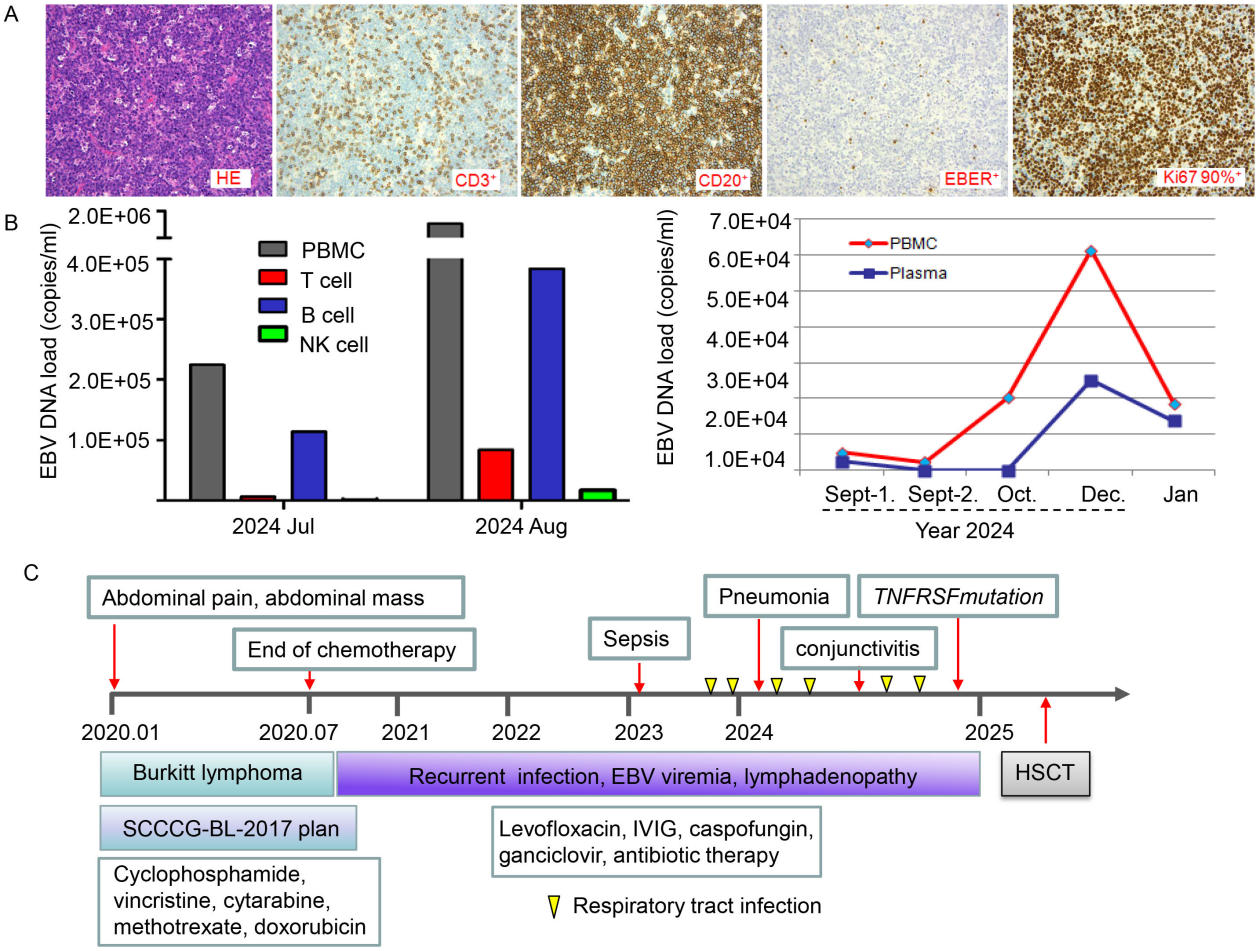
Clinical presentation and timeline of key clinical events. **(A)** Pathological and immunohistochemical characteristics at initial diagnosis of Burkitt’s lymphoma. CD20 positivity confirms B-cell origin, while CD3 negativity excludes T-cell origin. High Ki67 expression (near 100%) indicates a high proliferation rate, typical of Burkitt’s lymphoma. EBER positivity suggests Epstein-Barr virus (EBV) infection. **(B)** EBV sorting PCR to identify EBV-infected cell types and EBV load over time. EBV primarily infects B cells but can also infect T cells and NK cells, particularly in immunocompromised individuals. EBV DNA load (copies/mL) serves as an indicator of viral activity, disease progression, and treatment response. The graph illustrates changes in EBV load and cell type distribution throughout the disease course. **(C)** Timeline of key clinical events and laboratory test results, spanning from 2020 to 2025. Key events include symptoms (e.g., abdominal pain, abdominal mass, recurrent infections, EBV viremia, lymphadenopathy), complications (e.g., sepsis, pneumonia, conjunctivitis), and treatment milestones (e.g., chemotherapy completion, administration of cyclophosphamide, vincristine, cytarabine, methotrexate, doxorubicin, levofloxacin, IVIG, caspofungin, ganciclovir). Notable events such as TNFRSF variant and respiratory tract infections are highlighted.

This 9-year-old boy, born to consanguineous Chinese parents, was diagnosed with Burkitt’s lymphoma following the identification of an abdominal mass at the age of 4. The diagnosis of Burkitt’s lymphoma was confirmed by postoperative biopsy (**Figure 1A**). The patient was treated according to the SCCCG-BL-2017 regimen, including hormones, cyclophosphamide, vincristine, cytarabine, methotrexate, and doxorubicin. Chemotherapy ended after six months, and the patient has been under regular follow-up. Immunologic profiles of the patient were shown in **Table 1**. Over the past four years, he had recurrent respiratory infections, ear infections, hepatosplenomegaly, and lymphadenopathy. Laboratory tests revealed chronic EBV infection indicated numberous EBV+ B cells as well as EBV load in PBMC and plasma over time shown in **Figures 1B, C**, respectively. The patient had a 16-year-old sibling who succumbed to nasopharyngeal carcinoma (NPC). Due to recurrent infections and EBV viremia, the patient had undergone hematopoietic stem cell transplantation.

**Table 1:** Immunologic profiles of the patient.

Hemogram (normal range)	Ten years old (2025.01)	Nine years old (2024.08)	Five years old (2020.03)
WBCs (10^3^ cells/uL)	5.87	3.35	6.05
Hemoglobin (g/dL)	130	120	84
Neutrophils (10^3^ cells/uL)	2.18	1.00	3.83
Lymphocytes, (10^3^ cells/uL)	2.81	1.83	1.18
Monocytes (10^3^ cells/uL)	0.51	0.46	0.99
Platelets (10^3^ cells/uL)	126	115	134
Lymphocyte subsets			
CD3+% (38.56-70.06)	81.00	73.57	91.69
CD3+T (805-4459,/uL)	1591	680	1242
CD3+CD4+%(14.21-36.99)	39.07	38.34	22.28
CD3+CD4+T (345-2350,/uL)	807	366	314
CD3+CD8+% (13.24-38.53)	36.98	32.11	68.33
CD3+CD8+T (314-2080,/uL)	763	306	963
CD3+CD4+CD8+%	0.71	0.45	0.56
CD3+CD4+CD8+T (/uL)	15	4	8
CD19+% (10.86-28.03)	8.91	17.67	0.31
CD19+B (240-1317,/uL)	167	158	4
CD16+CD56+% (7.92-33.99)	7.20	6.93	7.24
CD16+CD56+ (210-1514,/uL)	134	62	94
CD4+CD25+Trag	39	24	69
IgG (5.96-13.64, g/L)	5.44	4.98	10.08
IgA 0.33-1.78, g/L)	<0.23	<0.23	0.29
IgM (0.52-2.42, g/L)	3.51	2.91	0.3

### Identification of a novel variant in *TNFRSF9* gene

**Figure 2:**
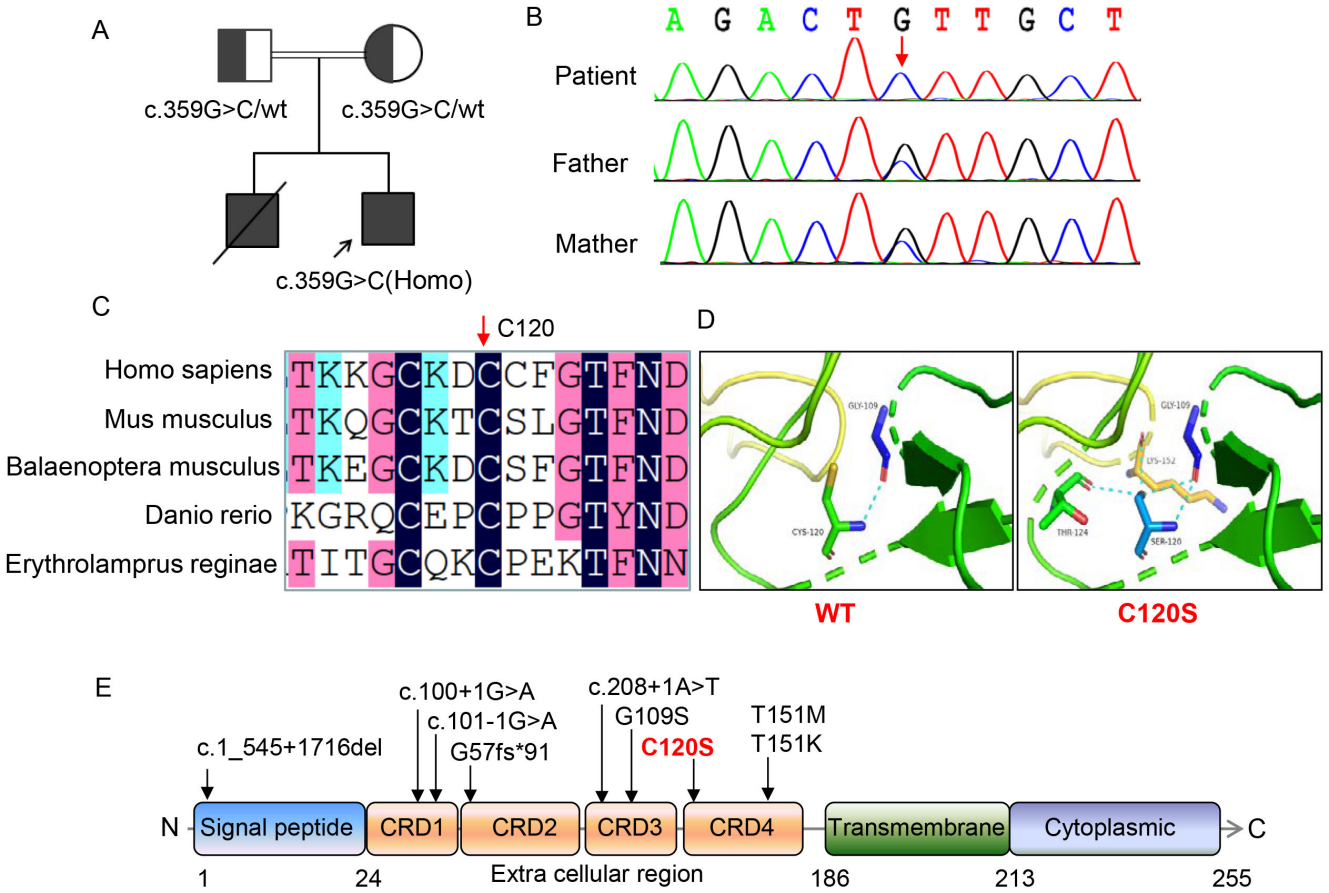
Genetic and molecular characterization of the patient with *TNFRSF9* variants. **(A)** Pedigrees of this family showing the inheritance pattern of the *TNFRSF9* c.359G>C variant. **(B)** Sanger sequencing of the *TNFRSF9* c.359G>C variant in this family, confirming the homozygous status of the c.359G>C variant in the patient and the heterozygous status in the parents. **(C)** Amino acid sequence alignment across species. The black bar indicated by red arrow highlights the highly conserved position of the 120^th^ residue in human emphasizing its evolutionary significance. **(D)** Structural impact of the C120S variant on protein stability. The variant introduces additional hydrogen bonds with neighboring residues (e.g., LYS-152 and THR-124), potentially affecting the protein’s conformation and function. **(E)** Distribution of *TNFRSF9* variants and protein domain structure. The variant studied in this work (C120S) is highlighted in red. The domain structure of the protein includes the signal peptide (amino acids 1-24), four extracellular cysteine-rich domains (25-186), including the ligand binding sites located in CRD2 and CRD3, transmembrane domain (187-213), and cytoplasmic region (214-255).

Trio-WES was performed to identify the genetic cause of the patient’s recurrent respiratory infections and EBV viremia, aiming to establish a definitive diagnosis. Bioinformatic analysis of the raw FASTQ sequencing data revealed a homozygous missense variant (c.359G>C, p.C120S) in the *TNFRSF9* gene (NM_001561.5). The variant was inherited in an autosomal recessive manner, with each parent contributing one allele (**Figures 2A, B**). The mutated site, located in extracellular region of the protein, is highly conserved across species, including mus musculus, balaenoptera musculus, danio rerio, and erythrolamprus reginae (**Figures 2C, E**). 3D structural analysis predicted reduced protein stability (ΔΔG -1.2189402) due to the formation of hydrogen bonds between the mutated S120 residue and nearby polar residues, potentially altering protein folding, structure, or stability (**Figure 2D**). This variant is absent from ClinVar, ExAC, gnomAD, and dbSNP databases. In silico predictions classified the variant as “disease-causing” (MutationTaster), “damaging or deleterious” (PROVEAN, SIFT, M-CAP, Polyphen, REVEL, and CADD), and “likely pathogenic” based on ACMG guidelines. No other pathogenic or likely pathogenic variants were identified in genes associated with EBV susceptibility or immunodeficiency-related lymphoproliferation. Combining the clinical presentations and genetic findings, a diagnosis of immunodeficiency with lymphoproliferation was confirmed.

### Functional CD8+ T-cell and B cell defects in *TNFRSF9*-deficient patient

**Figure 3:**
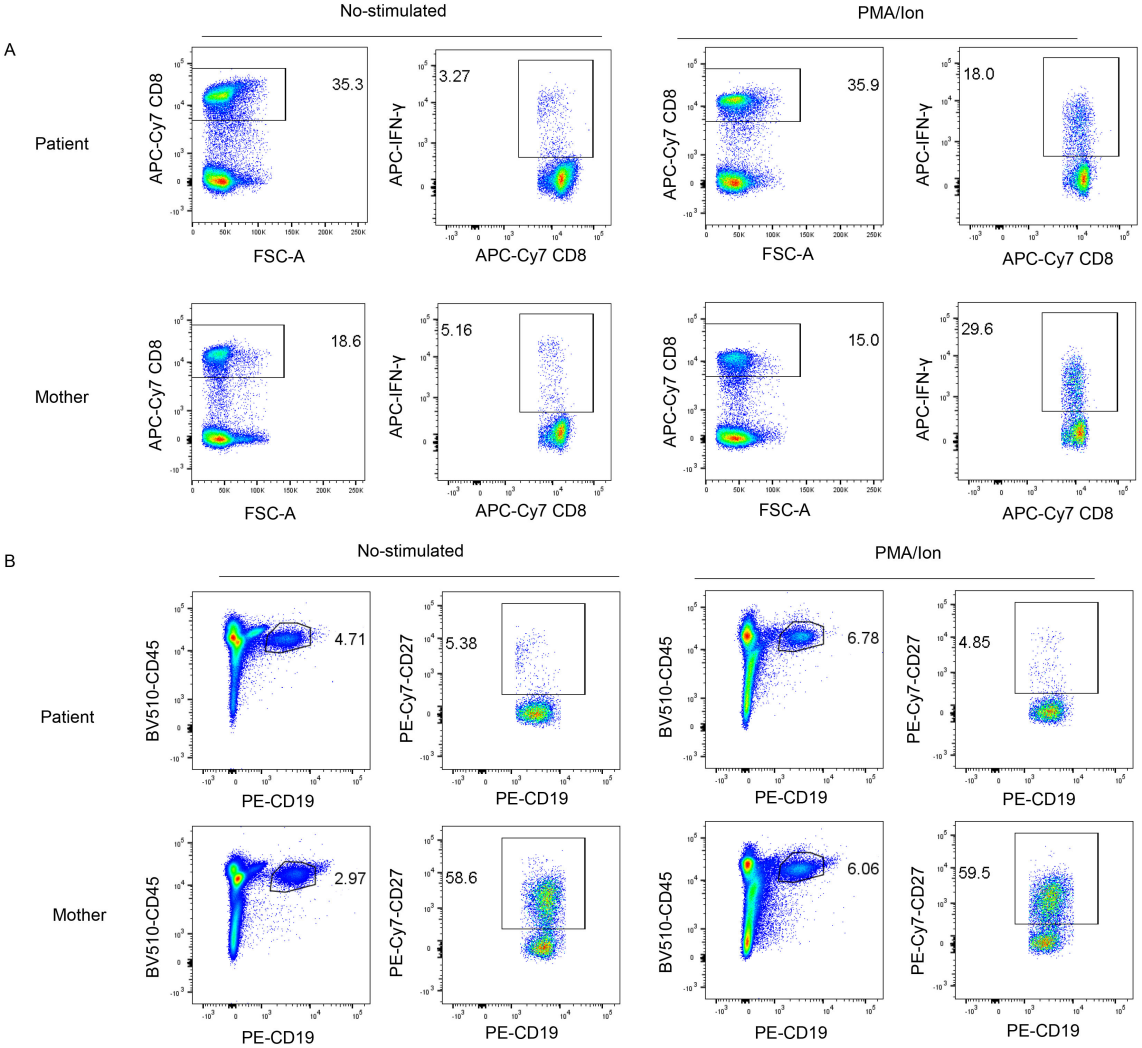
Immunological phenotypes of the patient. **(A)** Fluorescence-activated quantitative analysis of IFN-γ in CD8+T cells after stimulation with PMA/Ion, demonstrating a reduced release of IFN-γ in CD8+ T cells of the patient compared to the mother. **(B)** Peripheral blood B-cell immunophenotyping: Memory (CD19+ CD27+) frequencies in the patient and his mother, as measured by flow cytometry. The patient showed a lower frequency of CD27+ memory B cells compared to the mother, both under non-stimulated and stimulated conditions. These experiments were performed once and not repeated.

*TNFRSF9* costimulation had been showed to promote CD8+ cells to secret IFN-γ (6, 13), thus, we examined the release of IFN-γ under both non-stimulated and PMA (phorbol myristate acetate)/ionomycin-stimulated conditions, and found that the patient’s CD8+ T cells exhibited impaired IFN-γ release compared to the mother, suggesting a functional defect in CD8+ T-cell activity (**Figure 3A**). *TNFRSF9* is also expressed in activated B-cells and follicular dendritic cells, where it plays a key role in B-cell activation, affinity maturation and proliferation (12). The patient exhibited marked defects in B-cell maturation and differentiation, with a significant reduction in memory B cells (CD19+ CD27+) (**Figure 3B**). The frequency of CD27+ memory B cells was consistently lower in the patient than in the mother, indicating potential defects in memory B-cell function or quantity.

### Reduced expression of TNFRSF9, impaired AKT and NF-κB signaling *in vitro* cell model

**Figure 4:**
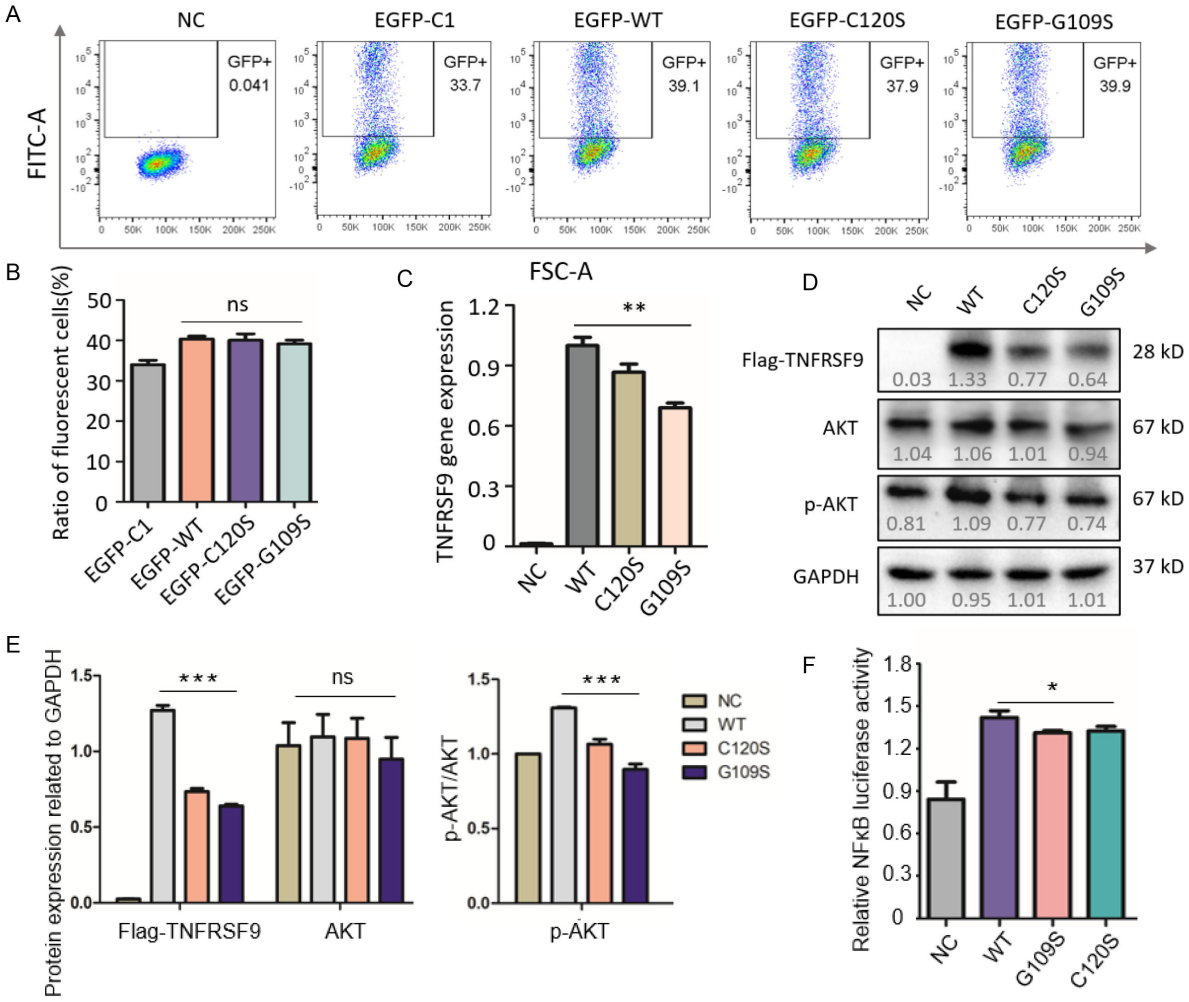
Functional analysis of the C120S variant in CD137 gene expression and signaling pathways. **(A, B)** Fluorescence intensity was analyzed by flow cytometry to evaluate cell transfection efficiency. **(C)** Expression levels of wild-type and mutant *TNFRSF9* in cells by real-time PCR. The WT group showed the highest expression level, while the C120S and G109S mutants exhibited slightly lower expression levels. **(D, E)** Western blot analysis of AKT and phosphorylated AKT (p-AKT) in cells transfected with wild-type or mutant *TNFRSF9* (including G109S as a positive control) and GAPDH was used as a loading control, and quantified by gray value using Image J software. The data indicate potential alterations in AKT signaling pathways due to the C120S mutation, as reflected by changes in the levels of total AKT and its phosphorylated form (p-AKT). **(F)** NF-kB-dependent luciferase activity in cell extracts from each sample, normalized to control cells transfected with WT-*TNFRSF9* plasmid. All experiments were repeated three times independently. One-way analysis of variance (ANOVA) was used when comparing three or more groups and statistical significance is indicated by *P<0.05; **P<0.01, ***P<0.001.

To investigate the effect of the variant (C120S), GFP fusion plasmids and flag-tagged plasmids encoding C120S, and WT *TNFRSF9*, as well as an empty vector, were transfected into 293T cells. Fluorescence intensity was analyzed by flow cytometry to evaluate cell transfection efficiency, and fluorescence signals were consistent indicating similar transfection efficiency (**Figures 4A, B**). Previous study has been showed that a homozygous variant, G109S, in *TNFRSF9* abolished protein expression, ligand binding, resulting in reduced proliferation of CD8+T cells, impaired expression of IFN-γ and perforin, and diminished cytotoxicity (9), thus, G109S was included as a positive control. Real-time PCR and Western blot analysis revealed significantly reduced mRNA and protein expression levels of the C120S and G109S variants compared to WT-*TNFRSF9* (**Figures 4C–E**). Additionally, phosphorylated AKT (p-AKT for pS473) levels were significantly decreased in cells with C120S or G109S variant, indicating the downregulation of the AKT signaling pathway (**Figure 4E**). These findings suggest that the C120S variant, similar to G109S, disrupted AKT signaling. Furthermore, luciferase assay demonstrated impaired NF-κB pathway activity (**Figure 4F**), highlighting its potential impact on NF-κB signaling activation.

### Literature review of cases with variants in the *TNFRSF9* gene

A literature review was conducted using databases such as PubMed, Medline, and ClinVar with the keyword “*TNFRSF9* gene”. We identified 4 articles reporting 9 cases (4, 9–11), and the clinical features of these patients, including our case, are summarized in **Table 2**. Among the 10 patients (7 males and 3 females; age ranging: 4-33 years), the most common clinical manifestations were recurrent infections (10/10), EBV viremia (10/10), hepatosplenomegaly (8/10), and lymphoma (4/10, including Burkitt and Hodgkin’s lymphoma). A total of 8 *TNFRSF9* variants have been identified, with most located in the extracellular region. 8 patients carried homozygous variants due to consanguineous marriages. Notably, patients within the same family, despite carrying the identical variants, exhibited significant variability in disease severity and symptom presentation.

**Table 2:** Clinical and genetic manifestations of *TNFRSF9*-deficient patients.

Patients	P1 (9)	P2 (9)	P3 (9)	P4 (9)	P5 (10)	P6 (10)	P7 (11)	P8 (11)	P9 (4)	P10	P11
Sex	M	M	M	M	F	M	M	F	F	M	M
Age	4 years	9 years	11 years	33 years	5 years	9 years	14 years	8 years	16 years	9 years	16 years
Age of onset	2 years	4 years	6 years	8 years	3 years	6 years	3 months	asymptomatic up to now	16 years	5 years	7years
Initial clinical manifestation	abdominal distention	recurrent respiratory infections	lower respiratory tract infection	recurrent infections	sinopulmonary infections, bronchiectasis, pneumococcal septicemia	recurrent sinopulmonary infections, generalized lymphadenopathy	recurrent upper respiratory, skin infections	asymptomatic	Recurrent sinopulmonary infections, fever	abdominal pain, abdominal mass	recurrent infections
Consanguinity	yes	yes	yes	no	yes	yes	yes	yes	no	yes	yes
Infectious complications during disease course	EBV viremia, recurrent ear infections	EBV viremia, recurrent pneumonia	EBV viremia, recurrent tonsillitis, recurrent otitis media, recurrent pneumonia	EBV viremia, recurrent pneumonia, pleuropneumonia	EBV viremia, pneumococcal septicemia; Sino pulmonary infections	EBV viremia, Sino pulmonary infections	episodes of panaritium, EBV viremia	EBV viremia	EBV+ LPD, chronic active Epstein–Barr virus, severe lung infections	EBV viremia; Recurrent respiratory infections; conjunctivitis	EBV viremia
Identified Pathogens	CMV, EBV	adenovirus, EBV, HSV	EBV, HSV	EBV	streptococcus pneumonia, EBV	EBV	Candida albicans, Staphylococcus aureus, HSV, EBV	no	EBV, Pseudomonas fluorescens, Candida albicans	EBV, mycoplasma, candida tropicalis, Staphylococcus aureus	NA
Hepatosplenomegaly	yes	yes	yes	yes	splenomegaly	splenomegaly	yes	no	splenomegaly	yes	NA
Lymphadenopathy	no	yes	yes	no	yes	yes	yes	no	yes	yes	NA
Malignancy	Burkitt lymphoma	no	EBV-positive Hodgkin’s lymphoma	no	no	EBV-positive Hodgkin´s lymphoma	no	no	no	Burkitt lymphoma	Nasopharyngeal carcinoma
Treatment of B-cell malignancies	cytoreductive prophase treatment: cyclophosphamide, dexamethasone, methotrexate	NA	etoposide, doxorubicin, vincristine	NA	NA	doxorubicin, bleomycin, vincristine, etoposide, prednisone, cyclophosphamide along with rituximab	NA	NA	NA	Cyclophosphamide, vincristine, cytarabine, methotrexate, doxorubicin	NA
Treatment of immunodeficiency/Infections	IVIG, co-trimoxazole, fluconazole	sirolimus, cellcept, co-trimoxazole	IVIG, antibiotic prophylaxis	Subcutaneous Immunoglobulin	IVIG, rituximab, stem cell transplantation	IVIG	Antibiotic intravenous infusion. prophylactic antibiotic treatment,	NA	intravenous ganciclovir, dexamethasone, bortezomib	IVIG, antibiotic intravenous infusion; antibiotic prophylaxis	NA
Other features	no	ALPS like symptoms, EBV associated lymphoproliferation	short stature	distal peptic esophagitis, erythematous gastritis	hemophagocytic lymphohystiocytosis	no	acute episode of HLH	no	no	Tic disorder	NA
Variant	c.1_545 + 1716del, Homo	c.452C>T (p.T151M), Homo	c.101 -1G>A, Homo	c.100 + 1G>A, Homo	c.325G>A (p.G109S), Homo	c.325G>A (p.G109S), Homo	c.170del, p.G57Vfs*56, Homo	c.170del, p.G57Vfs*56, Homo	c.208 + 1->AT, heter; c.452C>A (p.T151K), heter	c.359G>C, p.C120S	NA

## Discussion

EBV-specific immunity relies on robust cellular and humoral immune responses, with CD8+ cytotoxic T cells playing a critical role in controlling EBV infection. Patients with genetic variants affecting T-cell development and function are highly susceptible to chronic EBV infection (14). Growing evidence suggests that genetic defects impairing immune surveillance pathways increase susceptibility to EBV-associated lymphoproliferative disease (EBV^+^ LPD) (4). To date, over 20 monogenic disorders, including variants in *CD27*, *CD70*, *SH2D1A*, *ITK*, *MAGT1*, *PRKCD, PIK3CD, CORO1A*, *RASGRP1* and *CTPS1*, have been linked to immune dysregulation and susceptibility to EBV-driven B cell lymphoproliferative disorders (15–17). These defects impair virus-specific T-cell responses, highlighting critical pathways required for immune control of EBV. Research on these patients has offered valuable insights into the mechanisms of immune surveillance against EBV and the pathogenesis of EBV-driven malignancies.

In this study, we report a case presenting with EBV viremia, recurrent respiratory infections, and Burkitt lymphoma. By next-generation sequencing, a novel homozygous missense variant (c.359G>C, p.C120S) in the *TNFRSF9* gene was identified. Functional studies revealed that the p.C120S variant reduced *TNFRSF9* expression at both mRNA and protein levels, impaired AKT and NF-κB signaling pathways. These findings underscore the critical role of *TNFRSF9* in maintaining immune homeostasis and controlling EBV infection, and highlight the importance of molecular diagnostics in guiding targeted therapies for Inborn Errors of Immunity.

A review of the literature revealed only nine reported patients of *TNFRSF9* deficiency, with significant clinical heterogeneity even among patients carrying the same variant (**Table 2**). For example, Rodriguez et al. described two siblings from a consanguineous Pakistani family with a homozygous loss-of-function (LOF) variant c.170del in *TNFRSF9* (11). The brother had severe symptoms, including persistent fever, hepatosplenomegaly, recurrent lymphadenopathies, and high EBV load, culminating in a fatal acute episode of HLH at age 14. In contrast, his young sister remained asymptomatic despite persistent EBV viremia, except for abnormalities in memory B cell proportions. These findings suggest incomplete clinical penetrance or delayed disease onset in some patients with *TNFRSF9* variants. In our study, the patient had abdominal Burkitt’s lymphoma in at 4 years, followed by recurrent respiratory infections, ear infections, hepatosplenomegaly, lymphadenopathy, and chronic EBV infection. His elder brother had severer symptoms and succumbed to nasopharyngeal carcinoma (NPC), which has not been reported in other patients. Taken together, the clinical manifestation, severity and prognosis can be heterogeneous in patients with *TNFRSF9* variants.

Studies in *TNFRSF9*-deficient mice have demonstrated impaired T-cell survival, proliferation, and cytotoxicity, as well as defective NK-cell and cytotoxic T-lymphocyte (CTL) function (8, 12, 18). *TNFRSF9* is also essential for B-cell activation, proliferation, and class switch recombination (CSR) through its interaction with CD137L in germinal centers (12, 19, 20). In patients, EBV-specific CTL cytotoxicity was reduced, underscoring the role of *TNFRSF9* in controlling EBV infection and preventing lymphomagenesis (12). CD8+ T cells from affected patients showed impaired activation and reduced IFN-γ production. IFN-γ is essential for macrophage and NK-cell activation, antiviral and antitumor immunity, B-cell antibody production, Th1 differentiation, and memory T-cell proliferation. Its reduction likely contributes to weakened immune responses, increasing susceptibility to infections and impaired tumor surveillance. Additionally, a significant decrease in peripheral blood CD27+ memory B cells suggests an immunodeficient phenotype. In our patient, peripheral blood analysis revealed a significant decrease in CD27+ memory B cells was observed, and diminished IFNγ secretion by CD8+ T cells. A literature review of reported cases confirmed that all patients exhibited EBV viremia, with seven developing EBV-associated lymphoproliferative disorders and four diagnosed with lymphoma (4, 9–11). In our study, numberous EBV+ B cells were present in PBMC, in addition, we also observed a small number of EBV+ T and NK cells, indicating that EBV also infected these cells, though contamination could not be completely ruled out during the sorting of T cells or NK cells. Unfortunately, we did not test EBV by PCR for our patient after lymphocyte sorting during this period (between Oct 2024-Jan 2025), and we are unable to do this anymore as the patient has undergone hematopoietic stem cell transplantation. However, it is more likely that T and NK cells were infected by EBV, particularly in immunocompromised individuals, which had previously been in a patient with TNFRSF9 deficiency (4). These observations highlight *TNFRSF9* deficiency as a cause of immunodeficiency with a predisposition to lymphomagenesis. *TNFRSF9* is associated with various tumors, and somatic variants have been identified in hematopoietic, lymphoid, lung, and colorectal cancers (21–23). The COSMIC database lists 409 somatic variants of *TNFRSF9* (https://cancer.sanger.ac.uk/cosmic/gene/analysis?ln=*TNFRSF9* ), included in the C120S variant found in non-small-cell lung cancer (NSCLC), underscoring its relevance in tumorigenesis (24).

CD8+ T-cell function requires signals for survival, cell cycle progression, biomass formation, and differentiation into effector and memory cells. *TNFRSF9* is known to activate NF-κB signaling for cytokine induction and CD8+ T cell survival (25, 26). It triggers TRAF-dependent NF-κB activation to upregulate anti-apoptotic proteins, including Bcl-2 and Bcl-XL, while also promoting cell cycle progression via the PI3K and MEK-1/2 pathways (26). Additionally, NF-κB-dependent CD137 signaling regulates T-cell proliferation and differentiation (27). *TNFRSF9* activation has been shown to enhance CD8+ T cell proliferation by the TCF1/β-catenin axis via the PI3K/AKT/ERK pathway (28). In this study, we observed significantly reduced phosphorylated AKT levels and impaired NF-κB signaling *in vitro*, suggesting that the p.C120S variant disrupts these pathways, leading to abnormal CD8+T cell function. The observed defects in AKT and NF-κB signaling provide mechanistic insights into *TNFRSF9* deficiency, implicating these pathways in immune dysregulation, impaired antiviral responses, and increased lymphoma susceptibility.

*TNFRSF9* is a promising target for immunotherapy in both autoimmune diseases and malignancies. It functions as an immune suppressor by enhancing Treg expansion and mitigating T_H_17 -mediated autoimmunity, while also acting as a potent immune stimulator to enhance T-cell and NK-cell cytotoxicity and tumor infiltration (29, 30). Preclinical studies in mouse models have shown that anti-*TNFRSF9* agonist antibodies synergized with other therapies (31). Clinical trials of CD137 agonistic monoclonal antibodies and bispecific constructs are underway, demonstrating promising safety and efficacy (31, 32). Four patients with lymphoma responded well to chemotherapy and rituximab, maintaining stable conditions under IVIG and antibiotic prophylaxis (9). However, our patient experienced recurrent infections post-chemotherapy, underscoring the need for targeted immunotherapies.

In conclusion, this study identified a novel homozygous *TNFRSF9* variant (p.C120S) in a patient with EBV viremia, recurrent respiratory infections, and Burkitt lymphoma. Functional analyses revealed reduced *TNFRSF9* expression, impaired AKT and NF-κB signaling, and diminished CD8+ T-cell and memory B-cell function, highlighting the critical role of *TNFRSF9* in immune regulation and EBV control. These findings expand the genetic and clinical spectrum of *TNFRSF9*-related immunodeficiency and provide a foundation for developing targeted therapies. Further research is needed to elucidate the mechanisms underlying clinical heterogeneity and to optimize treatment strategies for affected patients.

## Data Availability

The datasets presented in this study can be found in online repositories. The names of the repository/repositories and accession number(s) can be found in the article/supplementary material.
